# Phylogenetic and Taxonomic Analyses of Five New Wood-Inhabiting Fungi of *Botryobasidium*, *Coltricia* and *Coltriciella* (Basidiomycota) from China

**DOI:** 10.3390/jof10030205

**Published:** 2024-03-08

**Authors:** Qian Zhou, Qianquan Jiang, Xin Yang, Jiawei Yang, Changlin Zhao, Jian Zhao

**Affiliations:** 1 College of Biodiversity Conservation, Southwest Forestry University, Kunming 650224, China; fungizq@163.com (Q.Z.); fungiqianquanjiang@163.com (Q.J.); fungixiny@163.com (X.Y.); 2 Office of Management and Protection, Green Peacock Provincial Nature Reserve, Dali 671000, China; 3 Yunnan Key Laboratory of *Gastrodia* and Fungal Symbiotic Biology, Zhaotong University, Zhaotong 657000, China; 4 Yunnan Academy of Biodiversity, Southwest Forestry University, Kunming 650224, China

**Keywords:** biodiversity, Botryobasidiaceae, Hymenochaetaceae, molecular systematics, multi-genes, taxonomy

## Abstract

In this present study, five new wood-inhabiting fungal taxa, *Botryobasidium gossypirubiginosum*, *Botryobasidium incanum*, *Botryobasidium yunnanense*, *Coltricia zixishanensis*, and *Coltriciella yunnanensis* are proposed. *Botryobasidium gossypirubiginosum* is distinguished by its slightly rubiginous hymenial surface, monomitic hyphal system, which branches at right angles, and subglobose, smooth basidiospores (14–17.5 × 13–15.5 µm); *B. incanum* is characterized by its white to incanus basidiomata having a hypochnoid hymenial surface, and ellipsoid, smooth basidiospores (6.5–8.5 × 3.5–5 µm); *B. yunnanense* is characterized by its buff to slightly yellowish hymenial surface, monomitic hyphal system, and broadly ellipsoid to globose, smooth, thick-walled basidiospores (11.5–14.5 × 9.5–10.5 µm); *Coltricia zixishanensis* differs in its rust brown pileal surface, and ellipsoid, thick-walled basidiospores (5–6.5 × 4–4.5 µm). *Coltriciella yunnanensis* is distinguished by its tiny pilei, short stipe, and navicular, verrucose basidiospores (10.5–12.5 × 6–7 µm). Sequences of ITS and *nLSU* genes were used for phylogenetic analyses using the maximum likelihood, maximum parsimony, and Bayesian inference methods. The phylogenetic results inferred from ITS sequences revealed that *B. gossypirubiginosum* was closely related to *B. robustius*; the species *B*. *incanum* was grouped with *B. vagum*; *B. yunnanense* was related to *B. indicum*. The species *C. zixishanensis* was grouped with *C. confluens* and *C. perennis*. ITS sequences revealed that *C. zixishanensis* was grouped into the genus *Coltriciella*, in which it was grouped with *Co. globosa* and *Co. pseudodependens*.

## 1. Introduction

Wood-inhabiting fungi are a vital component of forest ecosystems, playing several significant ecological roles [1,2]. They play a pivotal role in carbon storage and the regulation of nutrient cycling [3]. In fact, a variety of fungi, plants, and animals have different degrees of association with wood-inhabiting fungi, providing appropriate microenvironments for growth, reproduction, shelter and, sources of nutrients [4]. The genus *Botryobasidium* Donk (1931: 116) belonged to the family Botryobasidiaceae (Cantharellales, Basidiomycota), typified by *B*. *subcoronatum* (Höhn. & Litsch.) Donk (1931: 117) [5]. Based on the Index Fungorum (www.indexfungorum.org; accessed on 27 December 2023), the genus *Botryobasidium* has 106 specific and registered names with 78 species having been accepted worldwide [6]. Based on nLSU data analysis, this research demonstrated that the genus *Botryobasidium* formed a well-supported monophyletic group, as previously demonstrated by its micromorphological and ultrastructural characteristics [7,8].

The genus *Coltricia* Gray (1821: 644) is located in the family Hymenochaetaceae (Hymenochaetales, Basidiomycota), typified by *Coltricia perennis* (L.) Murrill (1903: 91) [9]. Based on the Index Fungorum (www.indexfungorum.org; accessed on 27 December 2023), the genus *Coltricia* has 129 specific and registered names, and currently 73 species have been accepted worldwide [10,11]. The genus *Coltriciella* Murrill (1904: 348) also belongs to the family Hymenochaetaceae (Hymenochaetales, Basidiomycota), typified by *C. dependens* (Berk. & M.A. Curtis) Murrill (1904: 348), and it is similar to *Coltricia* but is epixylous and has a vertically attached pileus [12]. Based on the Index Fungorum (www.indexfungorum.org; accessed on 27 December 2023), the genus *Coltriciella* has 23 specific and registered names, and currently 17 species have been accepted worldwide [12]. *Coltricia* and *Coltriciella* share similar morphological characteristics, but the latter is different in that it has smooth basidiospores [9,13,14]. Phylogenetically, *Coltricia* and *Coltriciella* comprise a monophyletic clade [15,16], but the previous study contended that phylogenetic analysis did not support the separation of the two genera [12,17]. Two new species of *Coltricia*, *C. subcinnamomea* L.S. Bian & Y.C. Dai and *C. subverrucata* L.S. Bian & Y.C. Dai, were described in China based on both morphological and molecular data, and the phylogenetic analyses based on ITS, nLSU, RPB2, and TEF1 data confirmed the generic positions of the two new species, *C. subcinnamomea* and *C. subverrucata* [18]. In recent research, tanalyses of rDNA ITS sequences supported the establishment of *Co. minuscula* Susan and Retn. & Sukarno, and the relationship between *Co. minuscula* and closely related species [19].

In this contribution, our main goal is to describe five new species collected from Yunnan Province, China, providing a detailed description of their morphology and molecular characterizations. We present the morphological characteristics and molecular analyses with ITS and nLSU DNA markers that support the taxonomy and phylogenetics of *Botryobasidium*, *Coltricia* and *Coltriciella* species.

## 2. Materials and Methods

### 2.1. Sample Collection and Herbarium Specimen Preparation

Fresh fruiting bodies of fungi growing on the branches and above-ground from angiosperms were collected in Qujing, Puer, Chuxiong, and Dali of Yunnan Province, China. The samples were photographed in situ, and fresh macroscopic details were recorded. Photographs were taken using a Jianeng 80D camera (Tokyo, Japan). All of the photos were focus-stacked and merged using Helicon Focus Pro7.7.5 software. Specimens were dried in an electric food dehydrator at 40 °C, then sealed and stored in an envelope bag, and deposited in the herbarium of the Southwest Forestry University (SWFC), Kunming, Yunnan Province, China.

### 2.2. Morphology

Our macroscopic morphological descriptions are based on field notes and photographs taken outdoors and in the laboratory, and follow Petersen’s color terminology [20]. The micromorphologic data of dried specimens were observed under a light microscope. The following abbreviations were used: KOH = 5% potassium hydroxide water solution; CB+ = cyanophilous; CB = cotton clue; CB− = acyanophilous; IKI = Melzer’s reagent; IKI− = both inamyloid and indextrinoid; L = mean spore length (arithmetic average for all spores); W = mean spore width (arithmetic average for all spores); Q = variation in the L/W ratios between the specimens studied; and n = a/b (number of spores (a) measured from a given number (b) of specimens).

### 2.3. DNA Extraction and Sequencing

The EZNA HP Fungal DNA Kit (Omega Biotechnologies Co., Ltd., Kunming, China) was used to extract DNA, with some modifications, from the dried specimens. The ITS and nLSU regions were amplified with the ITS5/ITS4 [21] and LR0R/LR7 [22] primer pairs, respectively. The PCR procedure for ITS and nLSU followed that in a previous study [22]. The PCR procedure for ITS was as follows: initial denaturation at 95 °C for 3 min, followed by 35 cycles at 94 °C for 40 s, at 58 °C for 45 s, and at 72 °C for 1 min, and a final extension of 72 °C for 10 min. The PCR procedure for nLSU was as follows: initial denaturation at 94 °C for 1 min, followed by 35 cycles at 94 °C for 30 s, at 48 °C for 1 min, and at 72 °C for 1.5 min, and a final extension of 72 °C for 10 min. The PCR products were purified and directly sequenced at Kunming Tsingke Biological Technology Limited Company, Yunnan Province, China. All of the newly generated sequences were deposited in GenBank (Table 1).

### 2.4. Phylogenetic Analyses

**Figure 1:**
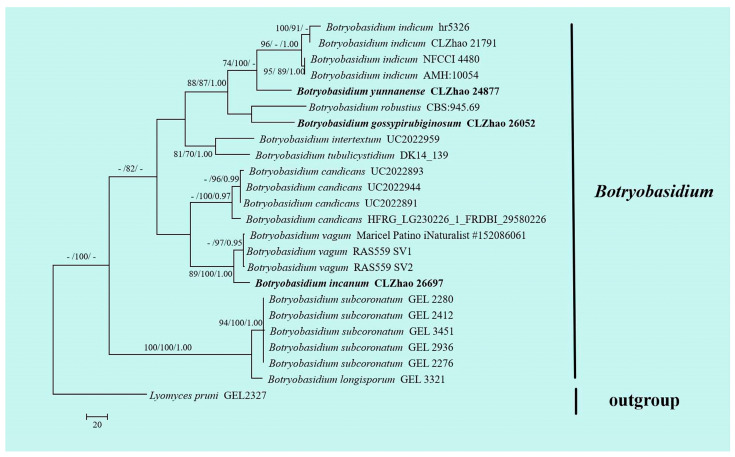
Maximum parsimony strict consensus tree illustrating the phylogeny of three new species and related species in *Botryobasidium* based on ITS sequences. Branches are labeled with maximum likelihood bootstrap values > 70%, parsimony bootstrap values > 50%, and Bayesian posterior probabilities > 0.95. The new species are in bold.

**Figure 2:**
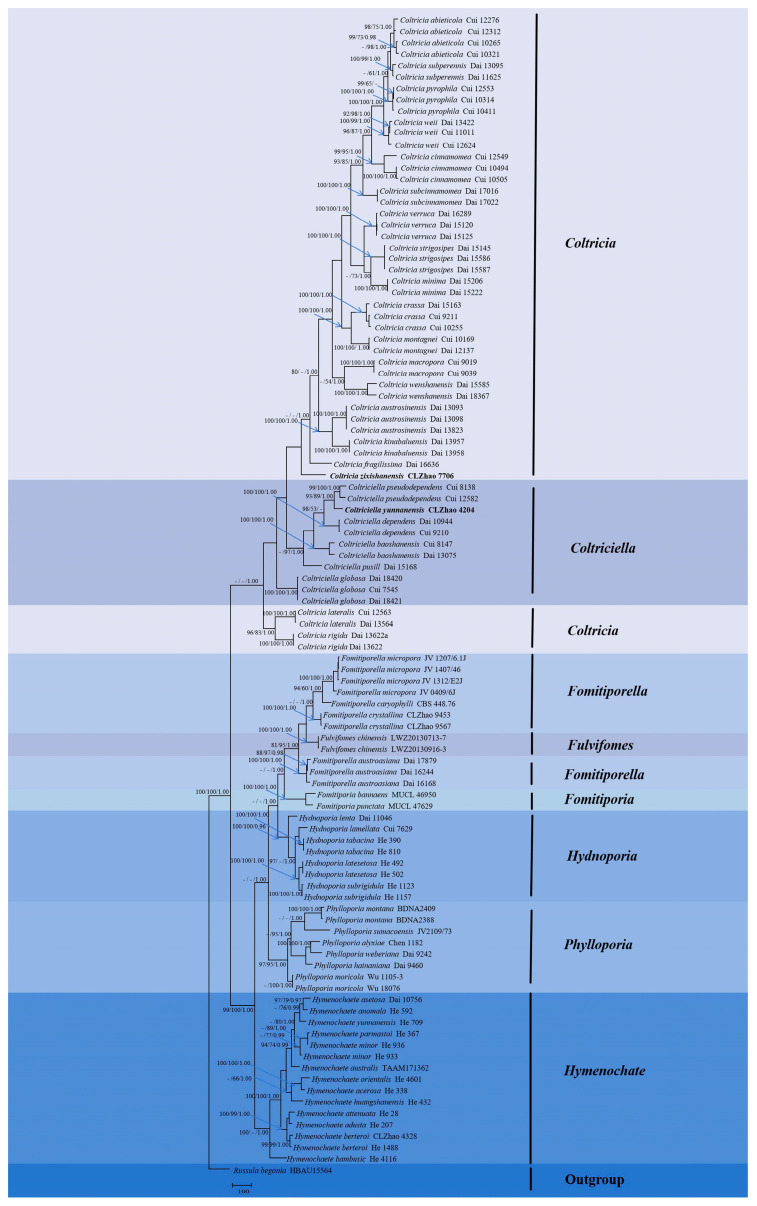
Maximum parsimony strict consensus tree illustrating the phylogeny of two new species of *Coltricia* and *Coltriciella* based on ITS + nLSU sequences. Branches are labeled with maximum likelihood bootstrap values > 70%, parsimony bootstrap values > 50%, and Bayesian posterior probabilities > 0.95. The new species are in bold.

**Figure 3:**
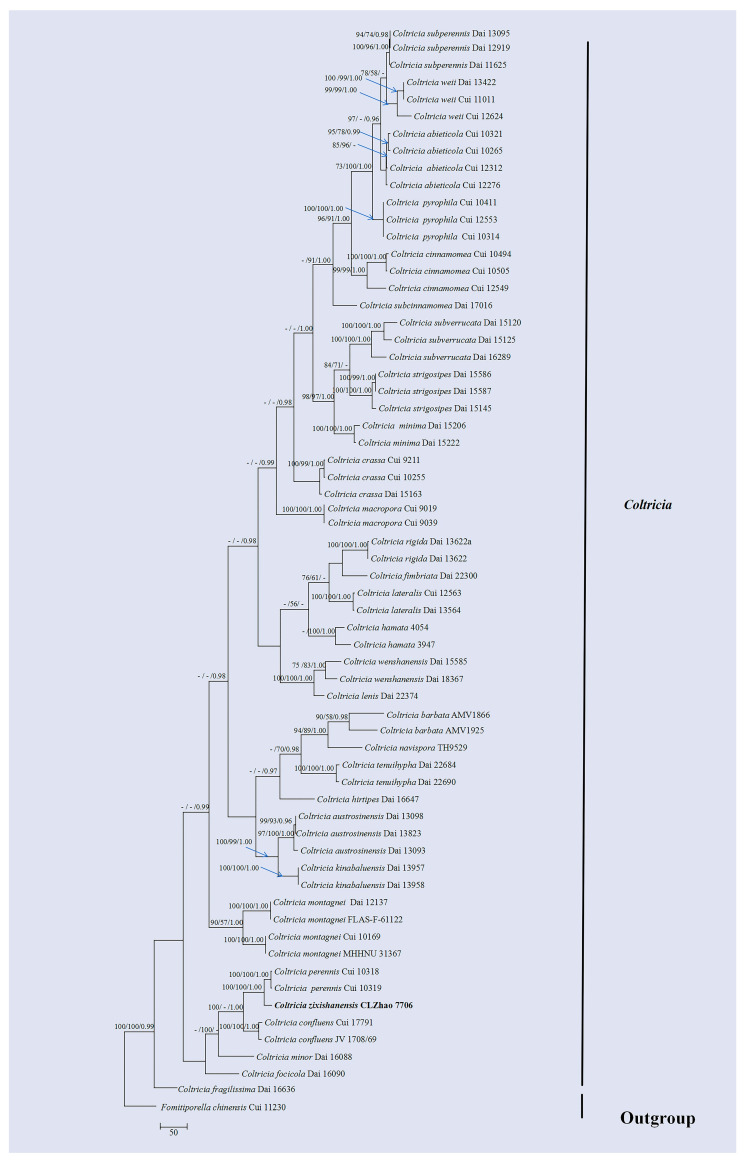
Maximum parsimony strict consensus tree illustrating the phylogeny of the *Coltricia zixishanensis* and related species in *Coltricia* based on ITS sequences. Branches are labeled with maximum likelihood bootstrap values > 70%, parsimony bootstrap values > 50%, and Bayesian posterior probabilities > 0.95. The new species are in bold.

**Figure 4:**
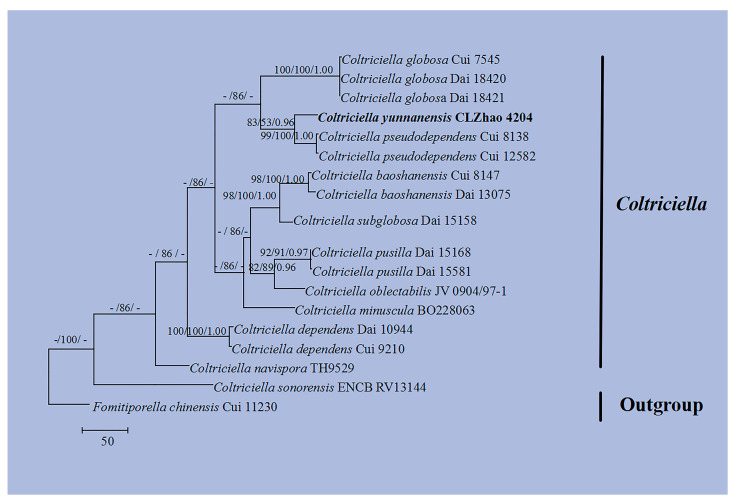
Maximum parsimony strict consensus tree illustrating the phylogeny of the Coltriciella yunnanensis and related species in *Coltriciella* based on ITS sequences. Branches are labeled with maximum likelihood bootstrap values > 70%, parsimony bootstrap values > 50%, and Bayesian posterior probabilities > 0.95. The new species are in bold.

The DNA sequences were aligned in MAFFT version 7 using the G-INS-i strategy [34]. The alignment was adjusted manually using AliView version 1.27 [35]. The sequence of *Lyomyces pruni* (Lasch) Riebesehl & Langer fetched from GenBank was used as an outgroup in ITS (Figure 1) analysis following a previous study’s analysis [31]. The sequence of *Russula begonia* G.J. Li, T.Z. Liu & T.Z. Wei retrieved from GenBank was used as an outgroup in ITS + nLSU (Figure 2) analysis following a previous study’s analysis [33]. The sequence of *Fomitiporia chinensis* (Pilát) Y.C. Dai, X.H. Ji & Vlasák retrieved from GenBank was used as an outgroup in ITS (Figure 3 and Figure 4) analysis following a previous study’s analysis [31].

Maximum parsimony (MP), maximum likelihood (ML), and Bayesian inference (BI) analyses were applied to the combined three datasets. Approaches to the phylogenetic analysis process followed those of Zhao and Wu [36]. MP analysis was performed in PAUP* version 4.0b10 [37]. All of the characters were equally weighted, and gaps were treated as missing data. Trees were inferred using the heuristic search option with TBR branch swapping and 1000 random sequence additions. Max-trees were set to 5000, branches of zero length were collapsed, and all most-parsimonious trees were saved. Clade robustness was assessed using bootstrap (BT) analysis with 1000 replicates [38]. Descriptive tree statistics, such as tree length (TL), the consistency index (CI), the retention index (RI), the rescaled consistency index (RC), and the homoplasy index (HI), were calculated for each most-parsimonious tree generated. ML was inferred using RAxML-HPC2 through Cipres Science Gateway (www.phylo.org (accessed on 10 January 2024)) [39]. Branch support (BS) for ML analysis was determined using 1000 bootstrap replicates and evaluated under the gamma model.

MrModeltest 2.3 [40] was used to determine the best-fit evolution model for each data set for Bayesian inference (BI), which was performed using MrBayes 3.2.7a with a GTR + I + G model of DNA substitution and a gamma distribution rate variation across sites [41]. Four Markov chains were run for 2 runs from random starting trees, for 1 million generations (Figure 1), 2 million generations (Figure 2), 1 million generations (Figure 3), and 2 million generations (Figure 4), and trees were sampled every 100 generations. The first one-fourth of all generations was discarded as a burn-in. The majority-rule consensus tree of all remaining trees was calculated. Branches were considered significantly supported if they received maximum likelihood bootstrap values (BS) > 70%, maximum parsimony bootstrap values (BT) >70%, or Bayesian posterior probabilities (BPP) > 0.95.

## 3. Results

### 3.1. Molecular Phylogeny

The dataset based on ITS (Figure 1) comprises sequences from 24 fungal samples representing 12 species. The dataset had an aligned length of 673 characters, of which 234 characters were constant, 89 characters were variable and parsimony-uninformative, and 350 characters were parsimony-informative. Maximum parsimony analysis yielded one equally parsimonious tree (TL = 935, CI = 0.7134, HI = 0.2866, RI = 0.8617, RC = 0.6147). Bayesian analysis and ML analysis resulted in a similar topology to that resulting from MP analysis with an average standard deviation of split frequencies = 0.004023 (BI), and the effective sample size (ESS) across the two runs was double the average ESS (avg ESS) = 1232.5.

The dataset based on ITS + nLSU (Figure 2) comprises sequences from 104 fungal specimens representing 56 species. The dataset had an aligned length of 2471 characters, of which 1097 characters were constant, 221 characters were variable and parsimony-uninformative, and 1153 characters were parsimony-informative. Maximum parsimony analysis yielded 35 equally parsimonious trees (TL = 7468, CI = 0.3502, HI = 0.6498, RI = 0.6488, RC = 0.2618). Bayesian analysis and ML analysis resulted in a similar topology to that resulting from MP analysis with an average standard deviation of split frequencies = 0.005527 (BI), and the effective sample size (ESS) across the two runs was double the average ESS (avg ESS) = 197.

The dataset based on ITS (Figure 3) comprises sequences from 67 fungal specimens representing 33 species. The dataset had an aligned length of 782 characters, of which 109 characters were constant, 153 characters were variable and parsimony-uninformative, and 520 were parsimony-informative. Maximum parsimony analysis yielded 216 equally parsimonious trees (TL = 3166, CI = 0.4166, HI = 0.5834, RI = 0.6414, RC = 0.2672). Bayesian analysis and ML analysis resulted in a similar topology to that resulting from MP analysis with an average standard deviation of split frequencies = 0.007467 (BI), and the effective sample size (ESS) across the two runs was double the average ESS (avg ESS) = 372.5.

The dataset based on ITS (Figure 4) comprises sequences from 18 fungal specimens representing 12 species. The dataset had an aligned length of 779 characters, of which 273 characters Were constant, 215 characters are variable and parsimony-uninformative, and 291 characters were parsimony-informative. Maximum parsimony analysis yielded two equally parsimonious trees (TL = 1044, CI = 0.7241, HI = 0.2759, RI = 0.6488, RC = 0.4698). Bayesian analysis and ML analysis resulted in a similar topology as MP analysis with an average standard deviation of split frequencies = 0.004052 (BI), and the effective sample size (ESS) across the two runs was double the average ESS (avg ESS) = 2563.5.

The phylogram based on the ITS rDNA gene regions (Figure 1) demonstrated that three new species were grouped into the genus *Botryobasidium*, in which *B. gossypirubiginosum* was closely related to *B. robustius* Pouzar & Hol.-Jech; *B*. *incanum* was grouped with *B. vagum* (Berk. & M.A. Curtis) D.P. Rogers; *B. yunnanense* was grouped with *B. indicum* (P.N. Singh & S.K. Singh) R. Kirschner & G. Langers. Based on the ITS and nLSU data (Figure 2), two genera, *Coltricia* and *Coltriciella*, clustered into the family Hymenochaetaceae Donk (Hymenochaetales, Agaricomycetes).The phylogram created based on inferences from the ITS data (Figure 3) showed that *C*. *zixishanensis* clustered into the genus *Coltricia*, in which it was grouped with two taxa, *C*. *confluens* P.J. Keizer and *C*. *perennis.* Based on the ITS data (Figure 4), *Co*. *yunnanensis* clustered into the genus *Coltriciella*, which was grouped with two taxa, *Co*. *globosa* L.S. Bian & Y.C. Dai and *Co*. *pseudodependens* L.S. Bian & Y.C. Dai.

### 3.2. Taxonomy

**Figure 5:**
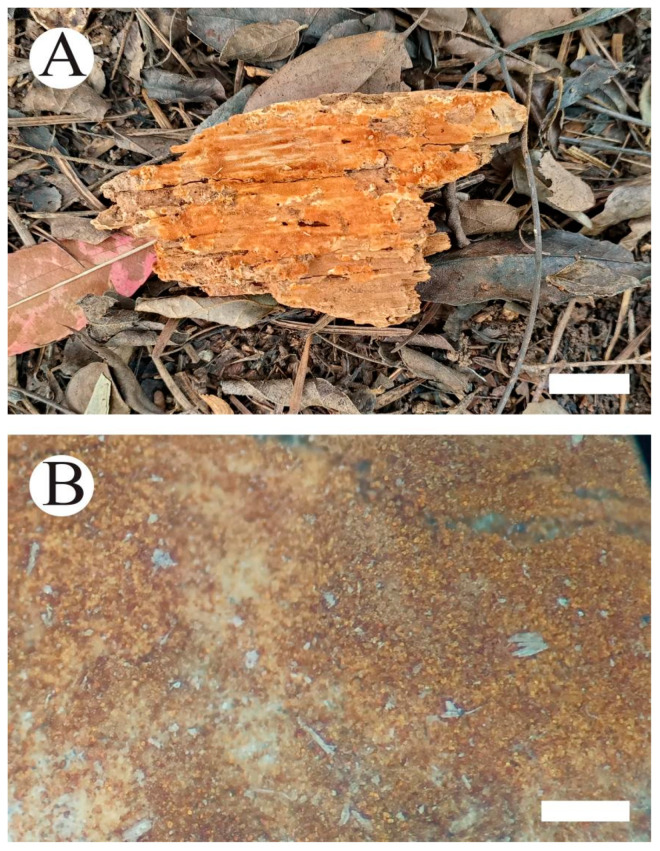
*Botryobasidium gossypirubiginosum*: basidiomata on the substrate (**A**); close up of the hymenophore (**B**). Bars: (**A**) = 1 cm and (**B**) = 0.5 mm.

**Figure 6:**
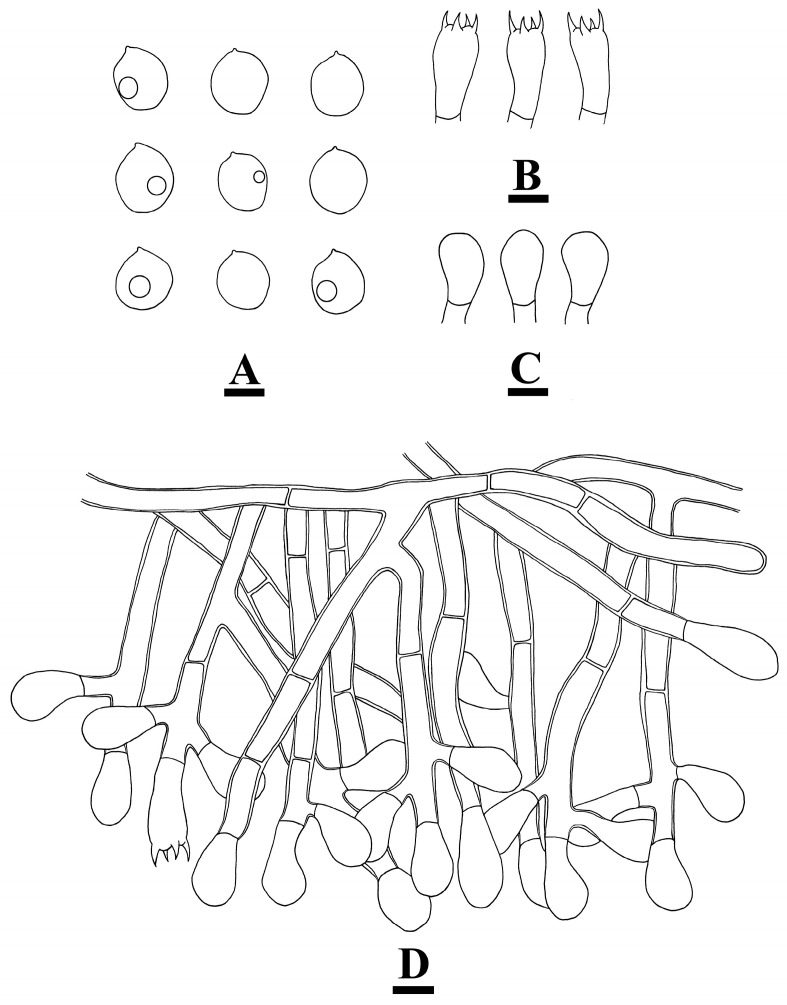
Microscopic structures of *Botryobasidium gossypirubiginosum*: basidiospores (**A**), basidia (**B**), basidioles (**C**), and a section of the hymenium (**D**). Bars: (**A**–**D**) = 10 µm.

***Botryobasidium gossypirubiginosum*** Q. Zhou & C.L. Zhao, sp. nov. Figure 5 and Figure 6.

MycoBank no.: MB851560

**Holotype**—China, Yunnan Province, Qujing, Qilin District, Cuishan Forest Park, GPS coordinates: 25°54′ N, 103°69′ E; altitude 2245 m asl., on fallen angiosperm branches, leg. C.L. Zhao, 6 November 2022, CLZhao 26,052 (SWFC).

**Etymology*—gossypirubiginosum*** (Lat.): from the Latin gossypium, referring to its cottony and rubiginous basidiomata surface.

**Basidiomata**—annual, resupinate. Hymenial surface floccose to cotton, slightly rubiginous when fresh, rubiginous on drying, up to 5 cm long, 3.5 cm wide, and 900 µm thick. Sterile margin indistinct, slightly rubiginous, and 1–2 mm wide.

**Hyphal system**—Monomitic, generative hyphae with simple septate, colorless, 6–8.5 µm wide, loosely interwoven, branched at right angles, basal hyphae thick-walled; IKI−, CB−, tissues unchanged in KOH.

**Hymenium**—Cystidia and cystidioles absent. Basidia clavate, in clusters on hymenial hyphal branches, with four sterigmata, and a base simple septate, 27.5–28 × 9.5–10 µm.

**Spores**—Basidiospores subglobose, smooth, yellowish, some with oil droplets inside, IKI−, CB+, (13.5−)14−17.5(−19) × (12−)13−15.5(−16) µm, L = 15.62 µm, W = 14.43 µm, Q = 1.08 (n = 30/1).

**Notes**—*Botryobasidium asperulum* (D.P. Rogers) Boidin, *B. danicum* J. Erikss. & Hjortstam, and *B. subcoronatum* (Höhn. & Litsch.) are similar to *B. gossypirubiginosum* in terms of them having a hypochnoid hymenial surface and thick-walled basal hyphae. However, *B. subcoronatum* is distinguishable from *B. gossypirubiginosum* through its yellowish to ochraceous hymenial surface, generative hyphae with clamp connections, basidia with six sterigmata, and smaller basidiospores (6–8 × 2.5–3 µm) [5]; *B. asperulum* is distinct from *B. gossypirubiginosum* in that it has smaller basidia (10–18 × 6–8 µm) with six sterigmata, and ellipsoid, smaller basidiospores (5–6 × 3–4 µm) [5]; *B. danicum* is distinct from *B. gossypirubiginosum* in that it has a greyish white to yellowish hymenial surface, and navicular and smaller basidiospores (12–14 × 3–5 µm) [5].

**Figure 7:**
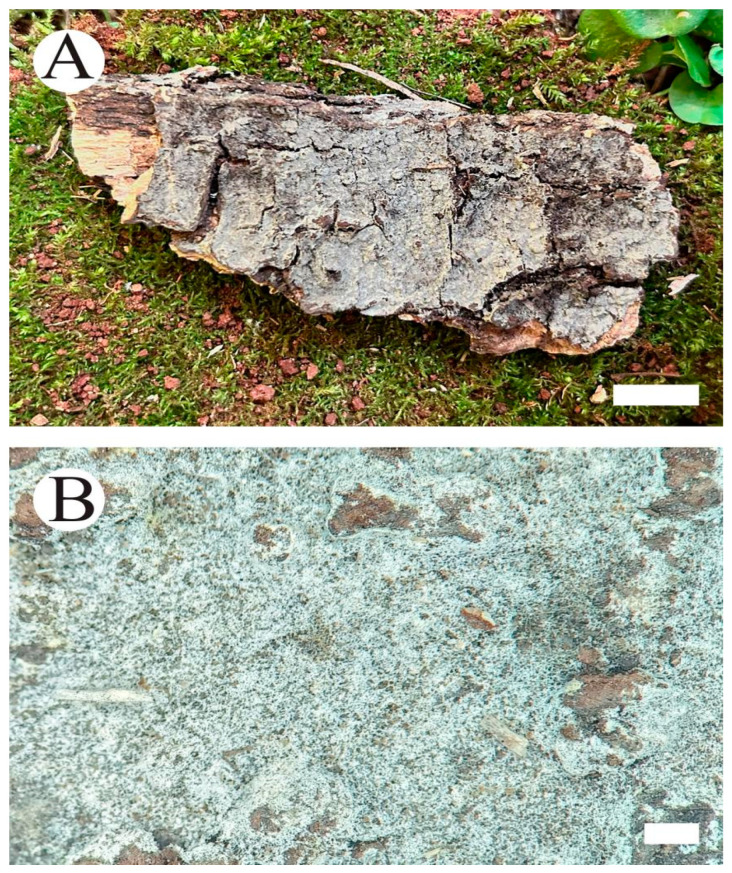
*Botryobasidium incanum*: basidiomata on the substrate (**A**); close up of the hymenophore (**B**). Bars: (**A**) = 1 cm and (**B**) = 1 mm.

**Figure 8:**
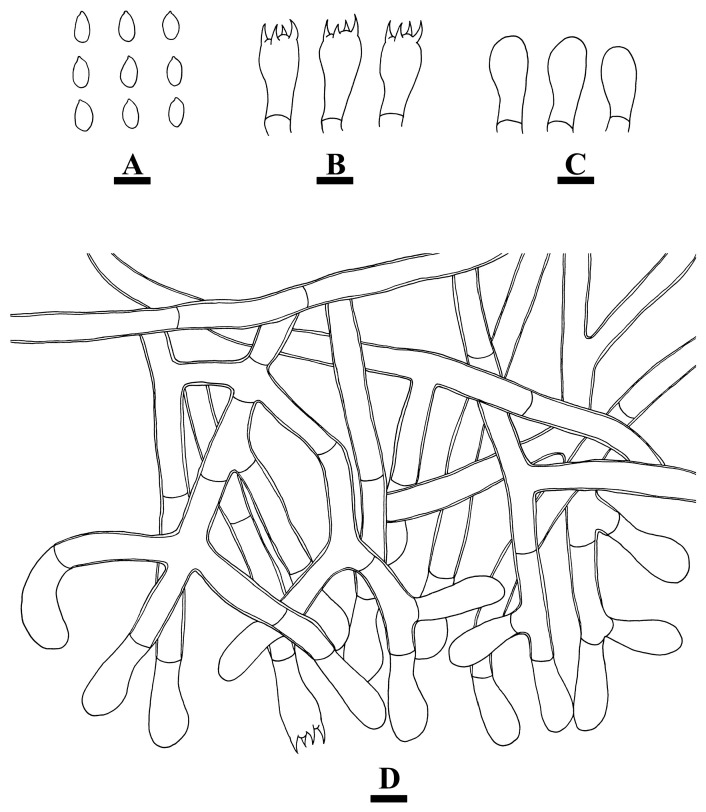
Microscopic structures of *Botryobasidium incanum*: basidiospores (**A**), basidia (**B**), basidioles (**C**), and a section of the hymenium (**D**). Bars: (**A**–**D**) = 10 µm.

***Botryobasidium incanum*** Q. Zhou & C.L. Zhao, sp. nov. Figure 7 and Figure 8.

MycoBank no.: MB851561

**Holotype**—China, Yunnan Province, Qujing, Qilin District, Cuishan, Forest Park, GPS coordinates: 25°54′ N, 103°69′ E; altitude 2245 m asl., on fallen angiosperm branches, leg. C.L. Zhao, 6 November 2022, CLZhao 26,697 (SWFC).

**Etymology**—***incanum*** (Lat.): referring to the incanus hymenial surface.

**Basidiomata**—Annual, resupinate, very thin, hypochnoid adnate, arachnoid, without odor or taste when fresh, up to 15 cm long, 5 cm wide, and 0.4 mm thick. Hymenial surface smooth, white to incanus when fresh, incanus on drying. Sterile margin indistinct, white to incanus, up to 0.5 mm wide.

**Hyphal system**—Monomitic, generative hyphae with simple septate, colorless, 8–10 µm wide, loosely interwoven, branched at right angles, basal hyphae thick-walled; IKI−, CB−, tissues unchanged in KOH.

**Hymenium**—Cystidia and cystidioles absent. Basidia clavate, in clusters on hymenial hyphal branches, with four sterigmata and a basal simple septate 23–25 × 6–7.5 µm.

**Spores**—Basidiospores ellipsoid, colorless, smooth, IKI−, CB− (5−)6.5–8.5(−9.5) × (3−)3.5–5(−5.5) µm, L = 7.48 µm, W = 4.23 µm, Q = 1.77 (n = 30/1).

**Notes**—*Botryobasidium candicans*, *B. pruinatum* (Bres.) J. Erikss and *B. sassofratinoense* Bernicchia & G. Langer are similar to *B. incanum* in that they have a hypochnoid hymenial surface. However, *B. candicans* is distinct from *B. incanum* in that it has thin-walled and subfusiform basidiospores [5]; *B. pruinatum* differs from *B. incanum* in that it has a greyish or yellowish to pale olivaceous hymenial surface and yellowish to brown generative hyphae, basidia with six slender sterigmata, and narrower basidiospores (5–8 × 2.5–3.5 µm) [5]; *B. sassofratinoense* is separated from *B. incanum* due to its whitish to pale ivory hymenial surface, generative hyphae with clamp connections, and navicular basidiospores [6].

**Figure 9:**
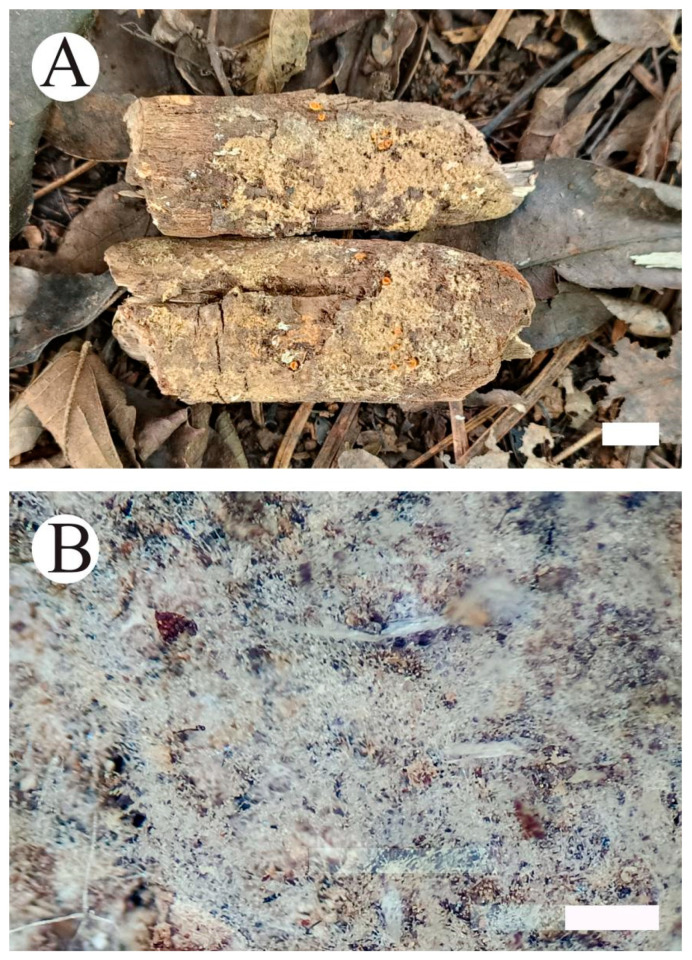
*Botryobasidium yunnanense*: basidiomata on the substrate (**A**); close up of the hymenophore (**B**). Bars: (**A**) = 1 cm and (**B**) = 0.5 mm.

**Figure 10:**
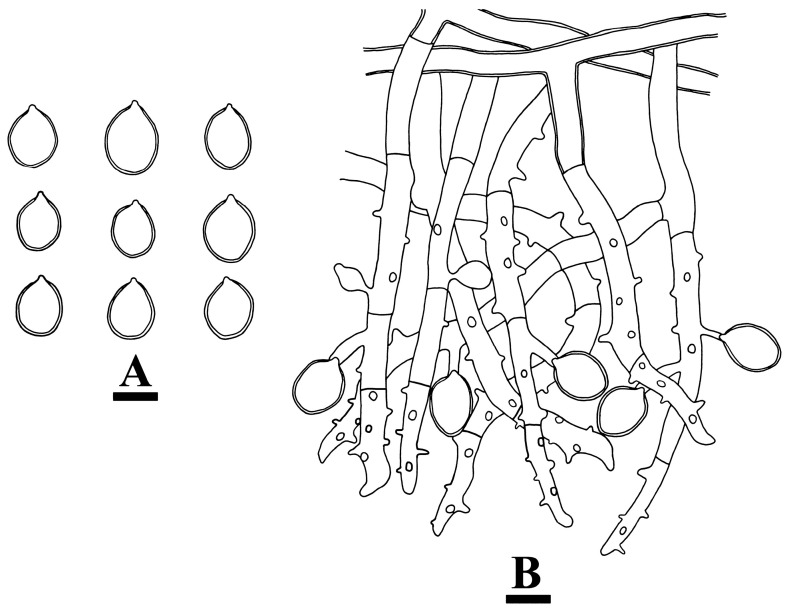
Microscopic structures of *Botryobasidium yunnanense*: basidiospores (**A**), a section of the hymenium with basidia, and basidioles and basidiospores (**B**). Bars: (**A**,**B**) = 10 µm.

***Botryobasidium yunnanense*** Q. Zhou & C.L. Zhao, sp. nov. Figure 9 and Figure 10.

MycoBank no.: MB851562

**Holotype**—China, Yunnan Province, Dali, Weishan County, Qinghua Town, GPS coordinates: 24°56′ N, 99°55′ E; altitude 2070 m asl., on fallen angiosperm branch, leg. C.L. Zhao, 18 October 2022, CLZhao 24,877 (SWFC).

**Etymology**—***yunnanense*** (Lat.): referring to the locality (Yunnan Province) of the type specimen.

**Basidiomata**—Annual, resupinate, very thin, hypochnoid. Hymenial surface floccose, buff to slightly yellowish when fresh, yellowish on drying, up to 8 cm long, 2.5 cm wide, and 100 µm thick. Sterile margin indistinct, buff to slightly yellowish, up to 1 mm wide.

**Hyphal system**—Monomitic, generative hyphae with simple septate, colorless, subhymenial hyphae 4–6 µm wide, basal hyphae 5.5–8 µm wide, slightly thick-walled, frequently branched at right angles; IKI−, CB−, tissues unchanged in KOH.

**Hymenium**—Cystidia and cystidioles absent. Basidia subcylindrical, 25–27 × 4.5–6 µm, with six sterigmata, simple septate at the base, basidioles similar in shape but slightly smaller.

**Spores**—Basidiospores broadly subglobose to globose, colorless, smooth, thick-walled, IKI−, CB−, (10.5−)11.5–14.5(−15.5) × (9−)9.5–10.5(−11.5) µm, L = 13.15 µm, W = 10.02 µm, Q = 1.31 (n = 30/1).

**Notes**—*Botryobasidium aureum* Parmasto, *B. conspersum* J. Erikss, *B*. *robustior* Pouzar & Hol.-Jech, and *B. medium* J. Erikss are similar to *B. yunnanense* in that they have basidia with six sterigmata [5]. The species *B. aureum* is separated from *B. yunnanens* due to it having a white to yellowish hymenial surface, and thin-walled, subcylindrical and smaller basidiospores (6–9 × 3–4 µm) [5]; *B. conspersum* is distinguished from *B. yunnanense* through its white to yellowish hymenial surface, and thin-walled, subcylindrical, and smaller basidiospores (7–9 × 2.5–3.5 µm) [5]; *B. medium* differs from *B. yunnanense* in that it has a whitish to pale-yellowish hymenial surface, basal hyphae with clamp connections, and navicular basidiospores [5]. *B*. *robustior* is different from *B. yunnanense* in that it has navicular to amygdaliform basidiospores [5].

**Figure 11:**
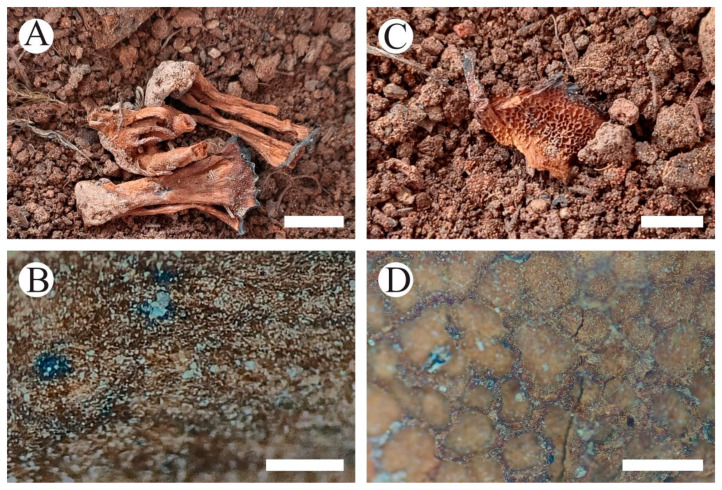
Basidiomata of *Coltricia zixishanensis*: the front of the basidiomata (**A**,**B**), the back of the basidiomata (**C**), and a section of the hymenophore (**D**). Bars: (**A**) = 0.5 cm; (**B**) = 1 mm; (**C**) = 0.5 cm; (**D**) = 1 mm.

**Figure 12:**
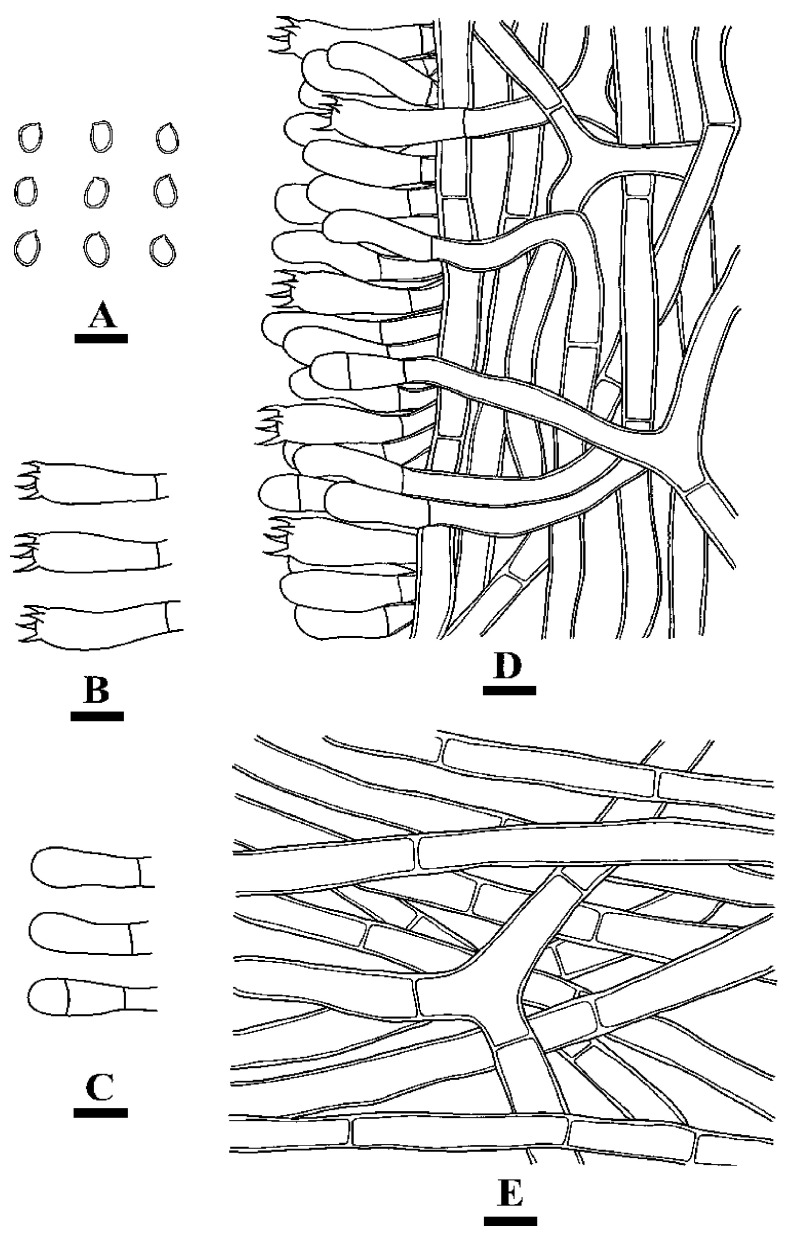
Microscopic structures of *Coltricia zixishanensis*: basidiospores (**A**), basidia (**B**), basidioles (**C**), part of the section of the hymenium (**D**), and hyphae from context (**E**). Bars: (**A**–**E**) = 10 µm.

***Coltricia zixishanensis*** Q. Zhou & C.L. Zhao, sp. nov. Figure 11 and Figure 12.

MycoBank no.: MB851563

**Holotype**—China, Yunnan Province, Chuxiong, Zixishan National Forest Park, GPS coordinates: 25°00′ N, 101°22′ E, altitude 2502 m asl., on the ground, leg. C.L. Zhao, 1 August 2018, CLZhao 7706 (SWFC).

**Etymology**—***zixishanensis*** (Lat.): referring to the locality (Zixishan National Forest Park) of the type specimen.

**Basidiomata**—Annual, centrally stipitate, solitary or adnate, without odor or taste when fresh, brittle and light-weight when dry. Pilei larger, circular, up to 1.5 cm in diameter and 1 mm thick at center, pilei surface rust brown, smooth, margin thin and sharp, roll inside when dry. Pore surface light brown, angular, 1–2 per mm, dissepiments thin, entire. Context rust brown, soft, spongy, up to 0.4 mm thick. Tubes dark brown, up to 0.6 mm thick. Stipe long, reddish brown, corky, up to 2.5 cm long, 4 mm in diameter.

**Hyphal system**—Monomitic, generative hyphae simple septate; tissue becoming blackish brown in KOH. Contextual hyphae yellowish, slightly thick-walled, branched, interwoven, 9.6–12.9 µm diameter. Tramal hyphae buff, thick-walled with a wide lumen, branched, frequently simple septate, straight, subparallel along the tubes, 7.1–10.2 μm in diameter.

**Hymenium**—Cystidia and cystidioles absent. Basidia clavate, with four sterigmata and a basal simple septate at the base, 22–29.5 × 7.5–10.5 µm; basidioles similar in shape but slightly smaller.

**Spores**—Basidiospores ellipsoid, colorless, thick-walled, smooth, IKI−, CB−, (4.5−)5–6.5(−7) × (3−)4–4.5(−5) µm, L = 5.72 µm, W = 4.24 µm, Q = 1.31–1.35 (n = 60/2).

**Notes**—*Coltricia abieticola* Y.C. Dai, *C. tenuihypha* L.S. Bian, M. Zhou & Jian Yu, and *C. wenshanensis* L.S. Bian & Y.C. Dai are similar to *C. zixishanensis* in that they have ellipsoid, thick-walled, and smooth basidiospores [27,29]. However, *C. abieticola* is distinguishable from *C. zixishanensis* through its smaller pores (2–4 per mm) and larger basidiospores (7–8 × 5.7–6.5 µm) [27,29]; *C. tenuihypha* is separated from *C. zixishanensis* due to its fan-shaped pilei, lacerate pileal margin, smaller pores (2–3 per mm), narrow and skeletal hyphae, and larger basidiospores (7.3–9.3 × 5.5–6.8 µm) [29]; and *C. wenshanensis* differs from *C. zixishanensis* in that it has larger basidiomata, with a distinctly concentrical and sulcate zonate, and larger basidiospores (7.5–8.2 × 6–6.8 µm) [25,27,28,42].

**Additional specimen examined (paratype)**—China, Yunnan Province, Chuxiong, Zixishan National Forest Park. GPS coordinates: 25°00′ N, 101°22′ E, altitude 2502 m asl., on the ground, leg. C.L. Zhao, 20 October 2023, CLZhao 35,615 (SWFC).

**Figure 13:**
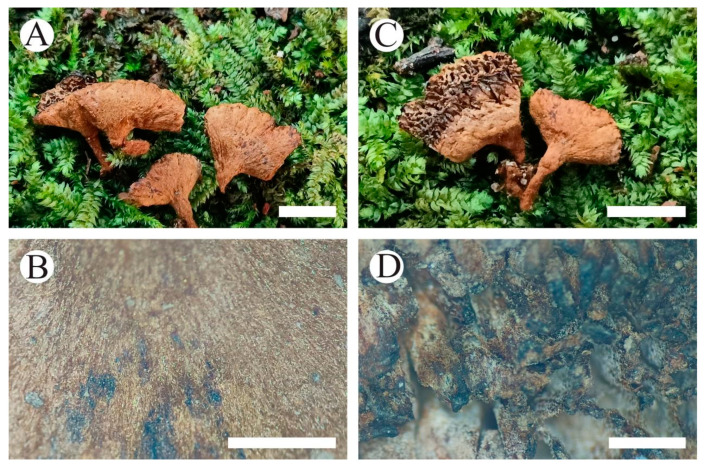
Basidiomata of *Coltriciella yunnanensis*: the front of the basidiomata (**A**,**B**), the back of the basidiomata (**C**), and a section of the hymenophore (**D**). Bars: (**A**) = 0.5 cm; (**B**) = 1 mm; (**C**) = 0.5 cm; (**D**) = 1 mm.

**Figure 14:**
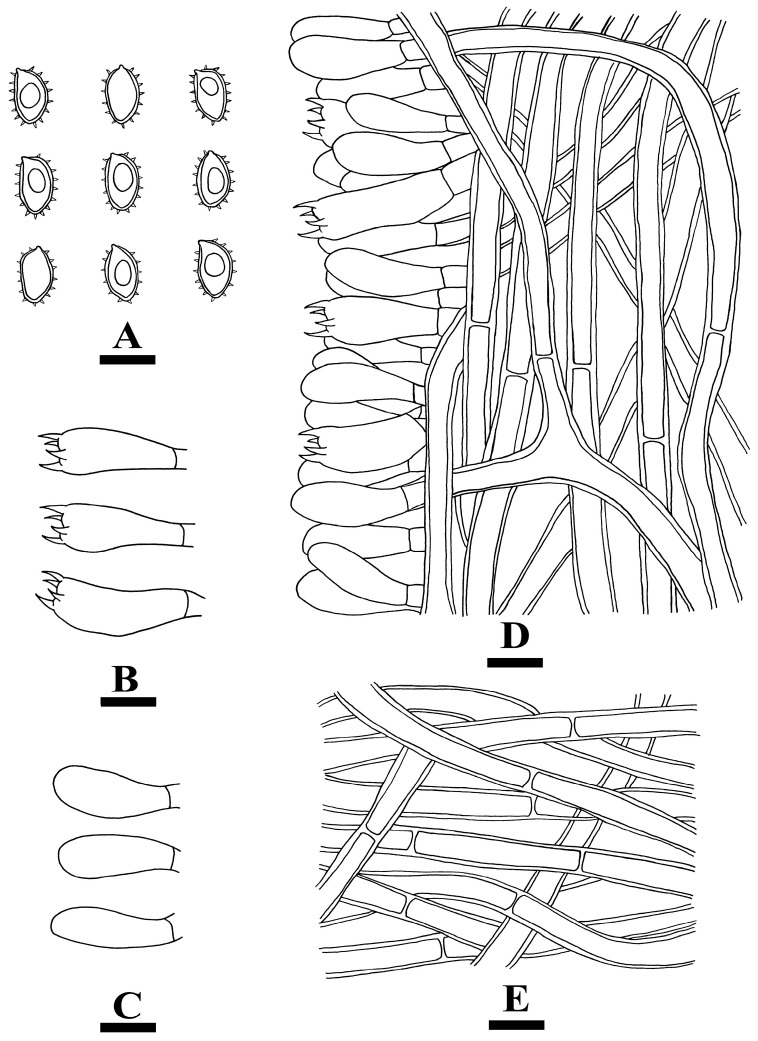
Microscopic structures of *Coltricia yunnanensis*: basidiospores (**A**), basidia (**B**); basidioles (**C**); part of the vertical section of the hymenium (**D**); hyphae from context (**E**). Bars: (**A**–**E**) = 10 µm.

***Coltriciella yunnanensis*** Q. Zhou & C.L. Zhao, sp. nov. Figure 13 and Figure 14.

MycoBank no.: MB851564

**Holotype**—China, Yunnan Province, Puer, Jingdong County, Wuliangshan National Nature Reserve, GPS coordinates: 23°57′ N; 100°22′ E, altitude 3300 m asl., on the ground, leg. C.L. Zhao, 5 October 2017, CLZhao 4204 (SWFC).

**Etymology**—***yunnanensis*** (Lat.): referring to the locality (Yunnan Province) of the type specimen.

**Basidiomata**—Annual, centrally stipitate, pendent, solitary or adnate, without odor or taste when fresh, becoming soft corky when dry. Pilei tiny, circular, up to 5 mm in diameter and 1 mm thick at center, fibrillose, hirsute, pilei surface fawn to grayish brown, margin thin and obtuse, curved down when dry. Pore surface light brown, angular, 1–3 per mm, dissepiments thin, entire. Context rust brown, soft, spongy, up to 0.4 mm thick. Tubes dark brown, up to 0.6 mm thick. Stipe short, reddish brown, corky, up to 4 mm long, 0.5 mm in diameter.

**Hyphal system**—monomitic; generative hyphae simple septate; IKI−, CB−, tissue darkening in KOH. Contextual hyphae yellowish-brown, thick-walled, occasionally branched, interwoven, 8–9.5 µm diameter. Tramal hyphae colorless, thick-walled with a wide lumen, rarely branched, frequently simple septate, straight, subparallel along the tubes, 8–9 μm in diameter.

**Hymenium**—Cystidia and cystidioles absent. Basidia broadly clavate, slightly sinuous, with four sterigmata and a basal simple septate at the base, 23.5–28 × 8.5–11 µm; basidioles similar in shape but slightly smaller.

**Spores**—Basidiospores navicular, golden brown, thick-walled, basidiospores finely verrucose, with oil droplets inside, IKI−, CB−, (10−)10.5–12.5(−13) × (5.5−)6–7 (−7.5) µm, L = 11.56 µm, W = 6.54 µm, Q = 1.77 (n = 30/1).

**Notes**—*Coltriciella baoshanensis* Y.C. Dai & B.K. Cui, *Co. corticicola* (Corner ex Y.C. Dai & Hai J. Li) Y.C. Dai & F. Wu, and *Co*. *oblectabilis* (Lloyd) Kotl., Pouzar & Ryvarden are similar to *Co. yunnanensis* in that they have golden-yellowish, thick-walled and finely verrucose basidiospores [18,42,43]. However, *Co. baoshanensis* is distinguishable from *Co. yunnanensis* through its conico-campanulate and tomentose pilei, hirsute stipe, short cylindricalbasidia with two sterigmata, and ellipsoid, smaller basidiospores (5.8–7.2 × 3.8–4.8 µm) [12]; *Co. corticicola* is separated from *Co. yunnanensis* due to its sessile basidiocarps with larger pilei, velutinate pileal surface, and mango-shaped basidiospores [43]; *Co*. *oblectabilis* differs from *Co. yunnanensis* in that it has ellipsoid and smaller basidiospores (8.5–10.2 × 5–5.9 µm) [43].

## 4. Discussion

In the several previous studies, molecular data confirmed phylogenetic relationships, in which the genus *Botryobasidium* nested in the cantharelloid clade, and was grouped with related genera: *Cantharellus*, *Craterellus*, *Hydnum*, and *Clavulina* [5,7]. Based on the molecular systematics study of *Coltricia* and *Coltriciella*, the result supported that both genera belonged to the family Hymenochaetaceae, and that both of them shared similar morphological features and a close molecular relationship [41,44].

In the present study, from the phylogram created based on inferences from the ITS data (Figure 1), three new species were grouped into the genus *Botryobasidium*, in which *B*. *gossypirubiginosum* clustered with *B. robustius*; *B. incanum* was closely related to *B. vagum*; *B. yunnanense* was grouped with *B. indicum*. From the molecular tree created based on inferences from the ITS + nLSU data (Figure 2), both genera, *Coltricia* and *Coltriciella*, clustered into Hymenochaetaceae. According to the ITS data (Figure 3), *C. zixishanensis* clustered into the genus *Coltricia*, in which it was grouped with two taxa, *C. confluens* and *C. perennis*. In the phylogram created based on inferences from the ITS data (Figure 4), *Coltriciella yunnanensis* clustered into the genus *Coltriciella*, in which it was grouped with two taxa, *Co. globosa* and *Co. pseudodependens*. However, morphologically, *B. robustius* differs from *B. gossypirubiginosum* in its smooth hymenophore and subnavicular to amygdaliform smaller basidiospores (7–9 × 3–4 µm) [5]; the species *B. vagum* is distinguished from *B. incanum* through its yellowish to greyish hymenial surface, basidia with six sterigmata, and navicular basidiospores [5]; *B. indicum* differs from *B. yunnanense* in yellow velvety hymenial surface and pyriform basidiospores [45]. *Coltricia confluens* is distinct from *C. zixishanensis* in that it expanded to having irregularly infundibuliform basidiomata, a distinct zonate, and larger basidiospores (7.1–8.5 × 4.6–5.2 µm); *C. perennis* is distinct from *C. zixishanensis* in that it has concentrical zonate basidiomata, a velutionous stipe, shorter basidia (16–20 × 6.5–8.5 µm), and longer basidiospores (6.5–9 ×4–5 µm) [29,46]. *Coltriciella globosa* differs from *Co. yunnanensis* in that it has greyish brown, velutinate basidiomata with a longer stipe, and globose basidiospores; *Co. pseudodependens* is distinct from *Co. yunnanensis* in that it has a concentrical zonate basidomata, pale-yellow contextual hyphae, smaller basidia (13–20 × 5–8 µm), and ellipsoid to oblong-ellipsoid basidiospores [47].

As wood-inhabiting fungi efficiently degrade lignocellulose in wood, they play a vital ecological role in the material circulation and energy flow of forest ecosystems, as well as leading to major economic value [46,48]. Therefore, they are important strategic biological resources [49,50]. Wood-inhabiting fungi are an extensively studied group of Basidiomycota, but their diversity is still unknown in China, where many of the recently described taxa of this ecogroup were found [51,52,53,54,55,56,57,58]. Based on morphological and molecular phylogenetic analysis, we described five new species from Yunnan Province, China. This study enriches our understanding of the diversity of wood-inhabiting fungi worldwide.

## Figures and Tables

**Table 1:** List of species, specimens, and GenBank accession numbers of sequences used in this study. New species are in bold.

Species Name	Sample No.	GenBank Accession No.		References
		ITS	nLSU	
*Botrybasidium candicans*	UC2022891	KP814227		[23]
*Botrybasidium candicans*	UC2022944	KP814546		[23]
*Botrybasidium candicans*	HFRC_LG230226_1_FRDBI_29580226	OR896129		Unpublished
*Botrybasidium candicans*	UC2022893	KP814200		[23]
***Botrybasidium gossypirubiginosum***	**CLZhao 26052**	OR668924	OR708665	**Present study**
***Botrybasidium incanum***	**CLZhao 26697**	OR668923	OR708664	**Present study**
*Botrybasidium indicum*	NFCCI 4480	NR171230		Unpublished
*Botrybasidium indicum*	AMH:10054	MK391496	MK391493	Unpublished
*Botrybasidium indicum*	hr5326	OP806032		Unpublished
*Botrybasidium indicum*	CLZhao 21791	ON406471		Unpublished
*Botrybasidium intertextum*	UC2022959	KP814540		Unpublished
*Botrybasidium longisporum*	GEL 3321	AJ389797		Unpublished
*Botrybasidium robustius*	CBS:945.69	MH859491	MH871272	[24]
*Botrybasidium subcoronatum*	GEL 2276	AJ389807		Unpublished
*Botrybasidium subcoronatum*	GEL 2280	AJ389806		Unpublished
*Botrybasidium subcoronatum*	GEL 2412	AJ389788		[24]
*Botrybasidium subcoronatum*	GEL 3451	AJ389796		[24]
*Botrybasidium subcoronatum*	GEL 2936	AJ389809		[24]
*Botrybasidium tubulicystidium*	DK14139	OL436769		Unpublished
*Botrybasidium vagum*	RAS559 SV1	OR471091		[18]
*Botrybasidium vagum*	RAS559 SV2	OR471092		Unpublished
*Botrybasidium vagum*	Maricel Patino iNaturalist#152086061	OR680661		Unpublished
***Botrybasidium yunnanense***	**CLZhao 24877**	OR668925	OR708666	**Present study**
*Coltricia abieticola*	Cui 10265	KX364784	KX364803	[25]
*Coltricia abieticola*	Cui 10321	KX364785	KX364804	[25]
*Coltricia abieticola*	Cui 12276	KU360673	KU360643	[25]
*Coltricia abieticola*	Cui 12312	KU360674	KU360644	[25]
*Coltricia austrosinensis*	Dai 13093	KU360670	KU360640	[25]
*Coltricia austrosinensis*	Dai 13098	KU360671	KU360640	[25]
*Coltricia austrosinensis*	Dai 13823	KU360672	KU360642	[25]
*Coltricia barbata*	AMV1866	KT724137		Unpublished
*Coltricia barbata*	AMV1925	KT724136		Unpublished
*Coltricia cinnamomea*	Cui 10494	KU360675	KJ000217	[25]
*Coltricia cinnamomea*	Cui 10505	KU360676	KU360645	[25]
*Coltricia cinnamomea*	Cui 12549	KY693728	KY693742	[25]
*Coltricia crassa*	Cui 9211	KU360677	KU360646	[25]
*Coltricia crassa*	Cui 10255	KU360678	KU360647	[25]
*Coltricia crassa*	Dai 15163	KU360679	KU360648	[25]
*Coltricia confluens*	Cui 17791	ON567327		Unpublished
*Coltricia confluens*	JV 1708/69	ON567325		Unpublished
*Coltricia fimbriata*	Dai 22300	NR182965		[26]
*Coltricia fragilissima*	Dai 16636	KY693733	KY693749	[25]
*Coltricia focicola*	Dai 16090	KX364786		[26]
*Coltricia hamata*	4054	MZ484546		[27]
*Coltricia hamata*	3947	MZ484545		[27]
*Coltricia hirtipes*	Dai 16647	KY693734	KU360649	[25]
*Coltricia kinabaluensis*	Dai 13957	KX364787	KX364806	[25]
*Coltricia kinabaluensis*	Dai 13958	KX364788	KX364807	[25]
*Coltricia lateralis*	Cui 12563	KX364789	KX364808	[25]
*Coltricia lateralis*	Dai 13564	KX364790	KX364809	[25]
*Coltricia lenis*	Dai 22374	OL691609	KJ000220	[26]
*Coltricia macropora*	Cui 9019	KU360680	KJ000221	[25]
*Coltricia macropora*	Cui 9039	KU360681	KU360649	[25]
*Coltricia minima*	Dai 15206	KU360682	KU360650	[25]
*Coltricia minima*	Dai 15222	KU360683	KJ000220	[25]
*Coltricia minor*	Dai 16088	KU360684		[28]
*Coltricia montagnei*	Cui 10169	KU360685	KU360652	[25]
*Coltricia montagnei*	Dai 12137		KX364810	[25]
*Coltricia montagnei*	MHHNU 31367	MK182316		Unpublished
*Coltricia montagnei*	FLAS-F-61122	MH399864		Unpublished
*Coltricia navispora*	TH9529	KT339262		Unpublished
*Coltricia perennis*	Cui 10318	KU360686	KU360650	[25]
*Coltricia perennis*	Cui 10319	KU360687	KU360652	[25]
*Coltricia perennis*	Cui 10318	KU360686		[28]
*Coltricia perennis*	Cui 10319	KU360687		[28]
*Coltricia pyrophila*	Cui 10314	KU360689	KU360655	[25]
*Coltricia pyrophila*	Cui 10411	KU360690	KU360656	[25]
*Coltricia pyrophila*	Cui 12553	KX364792	KX364812	[25]
*Coltricia rigida*	Dai 13622	KX364793	KX364813	[25]
*Coltricia rigida*	Dai 13622a	KX364794	KX364814	[25]
*Coltricia strigosipes*	Dai 15145	KX364795	KX364815	[25]
*Coltricia strigosipes*	Dai 15586	KU360692	KU360658	[25]
*Coltricia strigosipes*	Dai 15587	KU360693	KU360659	[25]
*Coltricia subcinnamomea*	Dai 17016	KY693740	KY693755	[25]
*Coltricia subcinnamomea*	Dai 17022		KY693756	[25]
*Coltricia subperennis*	Dai 11625	KY693735	KY693753	[25]
*Coltricia subperennis*	Dai 13095	KY693736	KY693754	[25]
*Coltricia subperennis*	Dai 12919	MT174242		[25]
*Coltricia tenuihypha*	Dai 22684	OL691610		[26]
*Coltricia tenuihypha*	Dai 22690	OL691611		[26]
*Coltricia verrucata*	Dai 15120	KU360694	KU360660	[25]
*Coltricia verrucata*	Dai 15125	KU360695	KU360661	[25]
*Coltricia verrucata*	Dai 16289	KU360696	KU360662	[25]
*Coltricia weii*	Cui 11011	KU360698	KU360664	[25]
*Coltricia weii*	Cui 12624	KX364796	KX364816	[25]
*Coltricia weii*	Dai 13422	KX364797	KX364817	[25]
*Coltricia wenshanensis*	Dai 15585	KX364798	KX364818	[25]
*Coltricia wenshanensis*	Dai 18367	MT174244	MT174237	[25]
***Coltricia zixishanensis***	**CLZhao 7706**	OR668922	OR708662	**Present study**
*Coltriciella baoshanensis*	Cui 8147	KX364799	KX364819	[25]
*Coltriciella baoshanensis*	Dai 13075	KX364800	KX364820	[25]
*Coltriciella dependens*	Dai 10944	KY693737	KY693757	[25]
*Coltriciella dependens*	Cui 9210	KY693738	KY693758	[25]
*Coltriciella globosa*	Cui 7545	KJ540930	KJ000226	[25]
*Coltriciella globosa*	Dai 18420	MT174245	MT174238	[25]
*Coltriciella globosa*	Dai 18421	MT174246	MT174239	[25]
*Coltriciella minuscula*	BO228063	KX086684		Unpublished
*Coltriciella navispora*	TH9529	KT339262		Unpublished
*Coltriciella oblectabilis*	JV 0904/97-1	ON567332		Unpublished
*Coltriciella pseudodependens*	Cui 8138	KJ540931	KJ000227	[25]
*Coltriciella pseudodependens*	Cui 12582	KX364801	KX364821	[25]
*Coltriciella pusilla*	Dai 15581	KY693739		[25]
*Coltriciella pusilla*	Dai 15168	KU360701	KU36066	[25]
*Coltriciella sonorensis*	ENCB RV13144	HQ439179		Unpublished
*Coltriciella subglobosa*	Dai 15158	KU360702		[25]
***Coltriciella yunnanensis***	**CLZhao 4204**	OR668921	OR708662	**Present study**
*Fomitiporella austroasiana*	Dai 16244	MG657328	MG657320	[29]
*Fomitiporella austroasiana*	Dai 16168	MG657329	MG657321	[29]
*Fomitiporella austroasiana*	Dai 17879	MG657330	MG657324	[29]
*Fomitiporella caryophylli*	CBS 448.76	AY558611	AY059021	[29]
*Fomitiporella chinensis*	Cui 11230	KX181309		[30]
*Fomitiporella crystallina*	CLZhao 9453	ON493552	ON493576	[29]
*Fomitiporella crystallina*	CLZhao 9567	ON493553	ON493577	[29]
*Fomitiporella micropora*	JV 1312/E2J	KX181294	KX181333	[29]
*Fomitiporella micropora*	JV 1407/46	KX181295	KX181332	[29]
*Fomitiporella micropora*	JV 0409/6J	KX181296	KX181331	[29]
*Fomitiporella micropora*	JV 1207/6.1J	KX181297	KX181330	[29]
*Fomitiporia bannaensis*	MUCL 46950	GU461943	EF429218	[31]
*Fomitiporia punctata*	MUCL 47629	GU461950	GU461982	[31]
*Fulvifomes chinensis*	LWZ20130713-7	KJ787817	KJ787808	[29]
*Fulvifomes chinensis*	LWZ20130916-3	KJ787818	KJ787809	[29]
*Hymenochaete acerosa*	He 338	JQ279543	JQ279657	[31]
*Hymenochaete adusta*	He 207	JQ279523	KU975497	[31]
*Hymenochaete anomala*	He 592	JQ279566	JQ279650	[31]
*Hymenochaete asetosa*	Dai 10756	JQ279559	JQ279642	[31]
*Hymenochaete attenuata*	He 28	JQ279526	JQ279633	[31]
*Hymenochaete australis*	TAAM171362	KM017414		[31]
*Hymenochaete bambusicola*	He 4116	KY425674	NG060687	[32]
*Hymenochaete berteroi*	CLZhao 4328	OM959409	OM967405	[28]
*Hymenochaete berteroi*	He 1488	KU975459	KU975498	[31]
*Hymenochaete huangshanensis*	He 432	NR120041	NG060638	Unpublished
*Hymenochaete minor*	He 933	NR120044	JQ279654	Unpublished
*Hymenochaete minor*	He 936	JQ279556		[31]
*Hymenochaete orientalis*	He 4601	KY425677	NG060688	[32]
*Hymenochaete parmastoi*	He 367	NR120102		[29]
*Hymenochaete yunnanensis*	He 709	JQ279571		Unpublished
*Hydnoporia lamellata*	Cui 7629	JQ279603	JQ279617	[30]
*Hydnoporia latesetosa*	He 492	JQ716404	JQ716411	[30]
*Hydnoporia latesetosa*	He 502	JQ716405	JQ716410	[30]
*Hydnoporia lenta*	Dai 11046	JQ279616	JQ279628	[30]
*Hydnoporia subrigidula*	He 1123	JQ716402	JQ716408	[30]
*Hydnoporia subrigidula*	He 1157	JQ716403	JQ716409	[30]
*Hydnoporia tabacina*	He 390	JQ279610	JQ279625	[30]
*Hydnoporia tabacina*	He 810	JQ279611	JQ279626	[30]
*Lyomyces pruni*	GEL2327	DQ340312		[31]
*Phylloporia alyxiae*	Chen 1182	LC528152	LC514407	[33]
*Phylloporia hainaniana*	Dai9460		JF712928	[27]
*Phylloporia montana*	BDNA2409	MH151177	MG738811	[27]
*Phylloporia montana*	BDNA2388	MH151176	MG738810	[27]
*Phylloporia moricola*	Wu 1105-3		LC514413	[27]
*Phylloporia moricola*	Wu 18076		LC589619	[27]
*Phylloporia sumacoensis*	JV2109/73	ON129552	ON006468	[27]
*Phylloporia weberiana*	Dai9242	LC528151	JF712936	[27]
*Russula begonia*	HBAU15564	MZ573252	OQ077072	[33]
*Lyomyces pruni*	GEL2327	DQ340312		[31]
*Phylloporia alyxiae*	Chen 1182	LC528152	LC514407	[33]
*Phylloporia hainaniana*	Dai9460		JF712928	[27]
*Phylloporia montana*	BDNA2409	MH151177	MG738811	[27]
*Phylloporia montana*	BDNA2388	MH151176	MG738810	[27]
*Phylloporia moricola*	Wu 1105-3		LC514413	[27]
*Phylloporia moricola*	Wu 18076		LC589619	[27]
*Phylloporia sumacoensis*	JV2109/73	ON129552	ON006468	[27]
*Phylloporia weberiana*	Dai9242	LC528151	JF712936	[27]
*Russula begonia*	HBAU15564	MZ573252	OQ077072	[33]
*Lyomyces pruni*	GEL2327	DQ340312		[31]
*Phylloporia alyxiae*	Chen 1182	LC528152	LC514407	[33]
*Phylloporia hainaniana*	Dai9460		JF712928	[27]
*Phylloporia montana*	BDNA2409	MH151177	MG738811	[27]

## Data Availability

Publicly available datasets were analyzed in this study. These data can be found through the following link: https://www.ncbi.nlm.nih.gov/; https://www.mycobank.org/page/Simple%20 names%20 search (accessed on 10 January 2024).
